# Identification of Hidden Cachexia Subgroup in PD‐L1‐High NSCLC: Comparative Analysis of the AWGC vs. Fearon Criteria

**DOI:** 10.1002/jcsm.70281

**Published:** 2026-04-12

**Authors:** Naoya Nishioka, Hayato Kawachi, Tadaaki Yamada, Motohiro Tamiya, Yoshiki Negi, Yasuhiro Goto, Akira Nakao, Shinsuke Shiotsu, Keiko Tanimura, Takayuki Takeda, Asuka Okada, Taishi Harada, Koji Date, Yusuke Chihara, Isao Hasegawa, Nobuyo Tamiya, Takeo Ogawa, Masahiro Iwasaku, Shinsaku Tokuda, Takashi Kijima, Koichi Takayama

**Affiliations:** ^1^ Department of Pulmonary Medicine, Graduate School of Medical Science Kyoto Prefectural University of Medicine Kyoto Kyoto Japan; ^2^ Department of Thoracic Oncology Osaka International Cancer Institute Osaka Osaka Japan; ^3^ Department of Respiratory Medicine and Hematology, School of Medicine Hyogo Medical University Nishinomiya Hyogo Japan; ^4^ Department of Respiratory Medicine Fujita Health University School of Medicine Toyoake Aichi Japan; ^5^ Department of Respiratory Medicine Fukuoka University Hospital Nanakuma Fukuoka Japan; ^6^ Department of Respiratory Medicine Japanese Red Cross Kyoto Daiichi Hospital Kyoto Kyoto Japan; ^7^ Department of Respiratory Medicine Japanese Red Cross Kyoto Daini Hospital Kyoto Kyoto Japan; ^8^ Department of Medical Oncology Fukuchiyama City Hospital Fukuchiyama Kyoto Japan; ^9^ Department of Respiratory Medicine Saiseikai Suita Hospital Suita Osaka Japan; ^10^ Department of Pulmonary Medicine Kyoto Chubu Medical Center Nantan Kyoto Japan; ^11^ Department of Respiratory Medicine Uji‐Tokushukai Medical Center Uji Kyoto Japan; ^12^ Department of Respiratory Medicine Saiseikai Shiga Hospital Ritto Shiga Japan; ^13^ Department of Respiratory Medicine Rakuwakai Otowa Hospital Kyoto Kyoto Japan

**Keywords:** Asian working group for cachexia, cachexia, fearon criteria, immunotherapy, non‐small cell lung cancer

## Abstract

**Background:**

Cancer cachexia, frequently observed in patients with non‐small cell lung cancer, is associated with reduced immunotherapy efficacy and poor prognosis. Despite the Fearon criteria being widely used to define cachexia, its relevance in Asian populations remains uncertain. Recently, the Asian Working Group for Cachexia (AWGC) proposed novel diagnostic criteria adapted for Asian body compositions. However, it remains unclear whether the outcomes or predictive value for immunotherapy differ between the AWGC and Fearon definitions. In addition, the AWGC‐specific cachexia subgroup, previously regarded as noncachexia under Fearon's definition, has not been fully characterized.

**Methods:**

We retrospectively analysed 411 Japanese patients with advanced PD‐L1–high non‐small cell lung cancer who received first‐line PD‐1/PD‐L1 monotherapy or chemoimmunotherapy. Survival outcomes and clinical and nutritional indices were compared using both the AWGC and Fearon criteria. The patients were subsequently classified into three groups: (1) noncachexia, (2) A‐only cachexia (meeting only the AWGC criteria), and (3) A+F cachexia (meeting both criteria). The small subgroup meeting only Fearon's criteria (*n* = 13) was excluded from analysis.

**Results:**

Per the AWGC definition, 168 patients (40.9%) were classified as having cachexia compared to 62 (15.1%) according to the Fearon definition. AWGC‐defined cachexia was associated with shorter overall survival (OS) (18.2 vs. 48.5 months, adjusted HR 1.539, *p* = 0.003), while progression‐free survival (PFS) under PD‐1/PD‐L1 inhibitor therapy or chemoimmunotherapy showed a nonsignificant trend (7.1 vs. 13.0 months, adjusted HR 1.253, *p* = 0.072). Fearon‐defined cachexia similarly predicted worse OS (18.4 vs. 37.7 months, adjusted HR 1.743, *p* = 0.002) but not PFS (5.9 vs. 11.4 months, adjusted HR 1.326, *p* = 0.081). In the three‐group comparisons, the A‐only cachexia group (*n* = 119) showed intermediate characteristics: Compared with the noncachexia group, they had lower BMI, higher C‐reactive protein and poorer nutritional indices (prognostic nutritional and geriatric nutritional risk indices); compared with the A+F cachexia group, they had higher BMI, lower C‐reactive protein and better nutritional indices. The PFS in the A‐only cachexia group was comparable with that in the noncachexia group; however, the OS was significantly shorter (21.8 vs. 48.5 months, *p* = 0.0002). Compared with the A+F cachexia group, the A‐only cachexia group showed no significant differences but demonstrated a favourable trend toward better PFS (adjusted HR 0.72, *p* = 0.096) and OS (adjusted HR 0.66, *p* = 0.051).

**Conclusion:**

The AWGC and Fearon criteria demonstrated comparable prognostic values. The A‐only cachexia subgroup, previously regarded as noncachexia, retained responsiveness to immunotherapy but exhibited significantly shorter survival, underscoring its clinical relevance as a previously unrecognized at‐risk group.

## 1 Introduction

Cancer cachexia is a complex syndrome involving progressive weight loss and disturbances in energy and protein metabolism [1]. More than half of patients with advanced lung cancer experience this condition, which is strongly associated with unfavorable outcomes [2, 3]. Reportedly, cachexia reduces the efficacy of both immune checkpoint inhibitors and chemoimmunotherapy, resulting in a worse prognosis [4, 5]. However, previous reports have all been based on the definition of cachexia proposed by Fearon et al. in 2011, which did not take into account differences in body size across ethnic groups [1, 6, 7]. Consequently, there has been growing concern that this definition may fail to accurately capture cachexia in Asian populations.

To overcome this limitation, the Asian Working Group for Cachexia (AWGC) developed new diagnostic criteria [8]. These criteria adopt lower thresholds for body mass index (BMI) and weight loss and include markers such as C‐reactive protein (CRP) and handgrip strength. This framework was developed not only to more appropriately reflect cachexia in Asian populations but also to facilitate its early detection. Therefore, the AWGC criteria are thought to include a population corresponding to the early stage of cachexia [1]. This stage has traditionally been difficult to distinguish from the non‐cachexia group and lacks standardized criteria. However, since the AWGC framework was designed for early detection, it may classify those with precachexia as having cachexia.

Using a cohort of Japanese patients with advanced lung cancer who received systemic therapy, including immunotherapy, this study aimed to clarify whether the prevalence of cachexia changes when adopting the AWGC criteria compared to the Fearon criteria and whether there are differences in treatment efficacy and prognosis. Furthermore, we sought to characterize the subgroup of patients newly classified as cachectic by the AWGC criteria but not by the Fearon definition and to evaluate their response to immunotherapy and overall prognosis.

## 2 Methods

### 2.1 Patients and Study Design

We performed a retrospective multicenter cohort study including non‐small cell lung cancer (NSCLC) patients who received at least one cycle of a programmed cell death protein 1 (PD‐1)/programmed cell death ligand 1 (PD‐L1) inhibitor (PD‐1/PD‐L1 inhibitor), either as monotherapy or in combination with chemotherapy, between March 2017 and December 2020. The study protocol was approved by the ethics committee of the Kyoto Prefectural University of Medicine and received additional approval from the institutional review boards of the 13 participating hospitals (approval no. ERB‐C‐2113). Eligibility criteria were (1) histological or cytological confirmation of NSCLC, (2) histologically proven Stages III–IV disease according to the American Joint Committee on Cancer eighth edition TNM classification or postoperative recurrence and (3) PD‐L1 expression level ≥ 50%. Clinical data were extracted from electronic medical records, and PD‐L1 status was determined via immunohistochemistry with a 22C3 pharmDx antibody (clone 22C3; Dako North America Inc., Carpinteria, CA).

### 2.2 Definition of Cachexia

Cachexia was defined according to both the AWGC and Fearon criteria. For the AWGC definition, cachexia was diagnosed when patients had either weight loss ≥ 2% within 6 months or a BMI < 21 kg/m^2^ regardless of weight loss, together with elevated CRP levels (> 0.5 mg/dL) as a marker of systemic inflammation. Handgrip strength and anorexia, included in the original AWGC criteria, could not be assessed in this retrospective study and were therefore excluded [8]. According to the Fearon definition, cachexia is defined as weight loss > 5% within 6 months or BMI < 20 kg/m^2^ with weight loss > 2% [1]. Sarcopenia, a part of the original Fearon criteria, could not be assessed in this study and was also excluded.

### 2.3 Outcome Assessment

The aims of this study were twofold: (1) to compare the differences and prognostic impact of cachexia, as defined by the AWGC and Fearon criteria, in patients with PD‐L1‐high NSCLC treated with first‐line PD‐1/PD‐L1 inhibitor monotherapy or chemoimmunotherapy and (2) to characterize the newly identified subgroup with cachexia using the AWGC criteria, clarifying its clinical features and treatment outcomes. Specifically, we evaluated the differences in progression free survival (PFS) and overall survival (OS) according to the presence or absence of cachexia, as defined by each criterion, and assessed whether there were marked differences in hazard ratios (HRs). Furthermore, using multivariate analysis, we compared the PFS and OS of patients newly classified as having cachexia (A‐only cachexia) to those of patients without cachexia (noncachexia: meeting neither the AWGC nor Fearon criteria) and to those with cachexia that met both criteria (A+F cachexia). In addition, we compared these groups in terms of body composition, inflammatory markers, and several nutritional indices. PFS was defined as the interval between the initiation of first‐line therapy and documented disease progression or death from any cause, whichever occurred first. OS was defined as the interval from the start of first‐line therapy to death from any cause. Survival data were censored at the cut‐off date of 28 February 2023, and the median follow‐up was 32.7 months.

### 2.4 Statistical Analysis

Categorical variables were compared using Fisher's exact test. Continuous variables, such as CRP and the nutritional indices, were analysed across the three groups using one‐way analysis of variance. Bonferroni correction was applied for post hoc pairwise comparisons between the groups to account for multiple testing. Survival was analysed using the Kaplan–Meier method, and differences between groups were assessed using the log‐rank test. A Cox proportional hazards model was used to examine the relationship between patient characteristics and survival. Based on the standard rule of at least 10 events per variable (EPV) rule [9], the observed event rates (74.5% for PFS and 53.8% for OS) were sufficient to include nine independent variables in the multivariate Cox model. The resulting EPV values were 34.0 for PFS and 24.6 for OS, both well above the recommended threshold.

Statistical analyses were performed using EZR (version 4.2; Saitama Medical Center, Jichi Medical University, Saitama, Japan), which is a graphical user interface for R (R Foundation for Statistical Computing) [10]. Statistical significance was set at *p* < 0.05.

## 3 Results

### 3.1 Patient Characteristics in the Overall Population

We retrospectively analysed 411 patients with advanced or recurrent NSCLC and PD‐L1 expression ≥ 50% who received immune checkpoint inhibitor monotherapy or chemoimmunotherapy between March 2017 and October 2021. The median patient age was 71 years (range, 36–90 years), and 77.9% of the patients were male. Most patients (86.1%) had an Eastern Cooperative Oncology Group performance status (ECOG PS) of 0–1. Histologically, adenocarcinoma was the most common subtype (58.2%), followed by squamous cell carcinoma (28.0%) and others (13.8%). The majority (81.5%) of patients had Stage IV disease at the time of treatment initiation, and 62.0% received immune checkpoint inhibitor monotherapy. Regarding pharmacological interventions for cachexia, anamorelin was administered to only one patient (0.3%) during the observed treatment period, indicating a negligible impact on the survival analysis. The median BMI was 21.4 kg/m^2^, and median CRP level was 1.4 mg/dL (Table S1).

### 3.2 Baseline Characteristics of the Cachexia and Noncachexia Groups Defined by the AWGC and Fearon Criteria

Cachexia was evaluated according to two distinct criteria, the AWGC and Fearon criteria, in the same cohort of 411 patients.

Based on the AWGC definition, 168 patients (40.9%) were diagnosed with cachexia. Compared to those without cachexia, they showed lower BMI (median 19.80 vs. 22.73 kg/m^2^, *p* < 0.001), greater weight loss (3.0% vs. 0.0%, *p* < 0.001) and higher CRP levels (3.20 vs. 0.41 mg/dL, *p* < 0.001). They were also more likely to have ECOG PS ≥ 2 (21.4% vs. 8.6%, *p* < 0.001), Stage IV disease (89.9% vs. 75.7%, *p* < 0.001) and liver metastases (20.8% vs. 8.6%, *p* = 0.001) but less likely to have brain metastases (11.3% vs. 19.8%, *p* = 0.029) (Table 1).

**TABLE 1:** Baseline characteristics of patients included in the study.

Characteristic	AWGC criteria			Fearon criteria		
	Cachexia (*n* = 168)	No cachexia (*n* = 243)	*p*	Cachexia (*n* = 62)	No cachexia (*n* = 349)	*p*
Age, y						
Median (range)	70 [36–90]	71 [40–88]	0.790	71 [40–85]	71 [36–90]	0.951
Sex						
Male	135 (80.4)	185 (76.1)	0.335	50 (80.6)	270 (77.4)	0.622
Female	33 (19.6)	58 (23.9)		12 (19.4)	79 (22.6)	
ECOG PS						
0–1	132 (78.6)	222 (91.4)	< 0.001	44 (71.0)	310 (88.8)	0.001
**≥** 2	36 (21.4)	21 (8.6)		18 (29.0)	39 (11.2)	
BMI (kg/m^2^)	19.80 [13.97–26.91]	22.73 [15.28–35.12]	< 0.001	17.84 [14.44–19.93]	22.14 [13.97–35.12]	< 0.001
Body weight loss (%)	3.0 [−3.0–23.0]	0.0 [−9.0–27.0]	< 0.001	7.0 [2.0–23.0]	0.0 [−9.0–27.0]	< 0.001
CRP (mg/dL)	3.20 [0.52–21.65]	0.41 [0.00–26.00]	< 0.001	3.01 [0.02–19.44]	1.13 [0.00–26.00]	0.002
Smoking history						
Yes	149 (88.7)	201 (82.7)	0.120	53 (85.5)	297 (85.1)	1.000
No	19 (11.3)	42 (17.3)		9 (14.5)	52 (14.9)	
Stage						
IV	151 (89.9)	184 (75.7)	< 0.001	53 (85.5)	282 (80.8)	0.478
Postoperative recurrence	17 (10.1)	59 (24.3)		9 (14.5)	67 (19.2)	
Histology						
Squamous cell carcinoma	58 (34.5)	57 (23.5)	0.026	13 (21.0)	102 (29.2)	0.347
Adenocarcinoma	85 (50.6)	154 (63.4)		41 (66.1)	198 (56.7)	
Others	25 (14.9)	32 (13.1)		8 (12.9)	49 (14.1)	
Driver gene alteration						
EGFR	6 (3.6)	8 (3.3)	1.000	6 (9.7)	8 (2.3)	0.01
ALK	2 (1.2)	4 (1.6)	1.000	0	6 (1.7)	0.597
ROS1	0	1 (0.4)	1.000	1 (1.6)	0	0.151
Liver metastasis	35 (20.8)	21 (8.6)	0.001	7 (11.3)	49 (14.0)	0.689
Brain metastasis	19 (11.3)	48 (19.8)	0.029	11 (17.7)	56 (16.0)	0.712
Anamorelin administration	1 (0.6)	0	0.469	0	1 (0.3)	1.000
Programmed cell death ligand 1, %						
50–89	102 (60.7)	157 (64.6)	0.467	36 (58.1)	223 (63.9)	0.394
90–100	66 (39.3)	86 (35.4)		26 (41.9)	126 (36.1)	
Treatment regimen						
Pembrolizumab monotherapy	102 (60.7)	153 (63.0)	0.680	37 (59.7)	218 (62.5)	0.673
Chemoimmunotherapy	66 (39.3)	90 (37.0)		25 (40.3)	131 (37.5)	

Abbreviations: ALK, anaplastic lymphoma kinase; AWGC, Asian Working Group for Cachexia; BMI, body mass index; CRP, C‐reactive protein; ECOG PS, Eastern cooperative oncology group performance status; EGFR, epidermal growth factor receptor; ROS1, Receptor Oncogene Serine/threonine kinase 1.

According to Fearon definition, 62 patients (15.1%) had cachexia. They also had lower BMI (17.84 vs. 22.14 kg/m^2^, *p* < 0.001), greater weight loss (7.0% vs. 0.0%, *p* < 0.001), higher CRP levels (3.01 vs. 1.13 mg/dL, *p* = 0.002) and were more likely to have ECOG PS ≥ 2 (29.0% vs. 11.2%, *p* = 0.001). However, no significant differences were observed in disease stage or incidence of liver or brain metastases. Notably, EGFR mutations were more common in the Fearon‐defined cachexia group (9.7% vs. 2.3%, *p* = 0.01), a difference that was not observed using the AWGC criteria (Table 1).

### 3.3 Overlap and Agreement Between the Cachexia Cohorts Defined by the AWGC and Fearon Criteria

**FIGURE 1:**
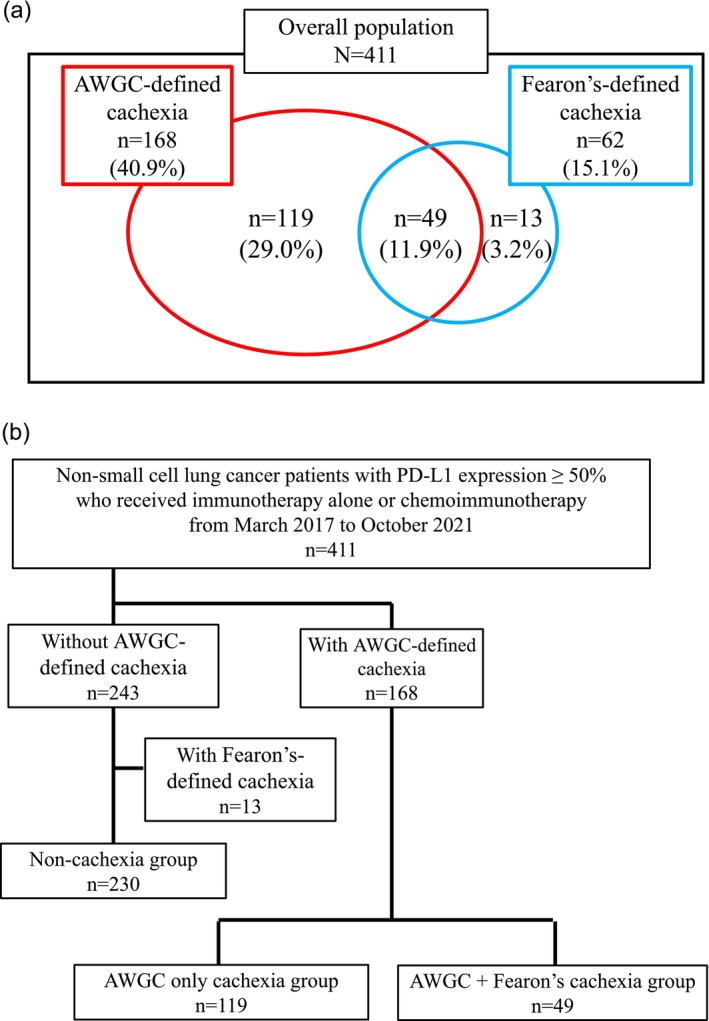
Classification of patients according to cachexia criteria. (a) Distribution of cachexia status based on the Asian Working Group for Cachexia (AWGC) and Fearon criteria in 411 non‐small cell lung cancer (NSCLC) patients with programmed cell death ligand 1 (PD‐L1) expression ≥ 50% treated with immunotherapy or chemoimmunotherapy. In total, 168 patients (40.9%) met the AWGC criteria and 62 patients (15.1%) met the Fearon criteria. (b) Patients were divided into three groups: non‐cachexia (*n* = 230); A‐only cachexia, defined as meeting only the AWGC criteria (*n* = 119); and A+F cachexia, defined as meeting both the AWGC and Fearon criteria (*n* = 49). In addition, 13 patients (3.2%) met only the Fearon criteria.

Of the 411 patients, 49 (11.9%) met both the AWGC and Fearon criteria, 119 (29.0%) met only the AWGC criteria and 13 (3.2%) met only the Fearon criteria (Figure 1a). Although most patients with Fearon‐defined cachexia were also identified using the AWGC definition, a substantial proportion of patients met only one of the two definitions.

Cohen's kappa coefficient for the agreement between the two definitions was 0.266, indicating limited concordance. This suggests that the AWGC and Fearon criteria identify partially overlapping but distinct clinical populations, likely because of differences in their diagnostic components.

### 3.4 Prognostic Impact of Cachexia Defined by the AWGC and Fearon Criteria

**FIGURE 2:**
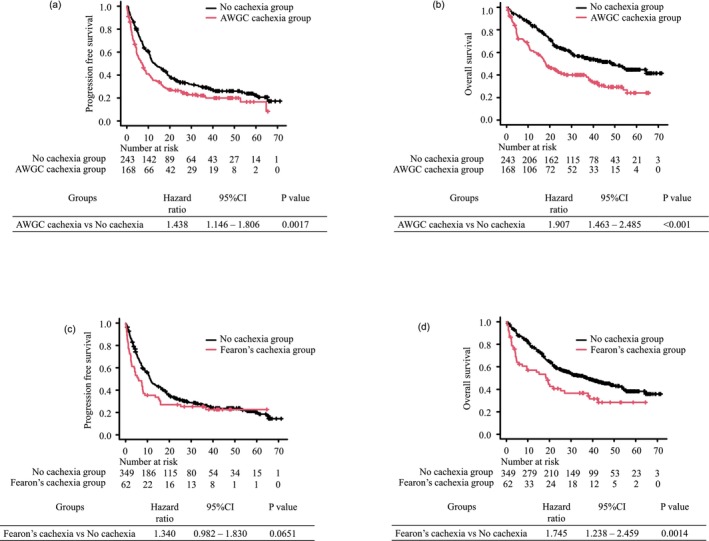
Kaplan–Meier survival curves comparing the cachexia and non‐cachexia groups defined by the Asian Working Group for Cachexia (AWGC) and Fearon criteria. (a) Progression‐free survival (PFS) according to AWGC‐defined cachexia status. (b) Overall survival (OS) according to AWGC‐defined cachexia status. (c) PFS according to Fearon‐defined cachexia status. (d) OS according to Fearon‐defined cachexia status.

As of the cutoff date, with a median follow‐up of 32.7 months, disease progression and death were observed in 306 (74.5%) and 221 (53.8%) of the 411 patients, respectively. In the overall population, AWGC‐defined cachexia was significantly associated with shorter PFS and OS (median PFS: 7.1 vs. 13.0 months, *p* = 0.001; median OS: 18.2 vs. 48.5 months, *p* < 0.001) (Figure 2a,b). In the multivariate Cox regression analysis adjusted for age, ECOG PS, histology, disease stage, PD‐L1 status, presence of metastases and treatment regimen, the association with OS remained significant (adjusted HR: 1.539, 95% confidence interval [CI]: [1.156–2.048], *p* = 0.003), whereas PFS showed a trend toward shorter survival that did not reach statistical significance (adjusted HR: 1.253, 95% CI: [0.980–1.602], *p* = 0.072) (Table 2).

**TABLE 2:** Univariate and multivariate analyses of PFS and OS according to the AWGC cachexia criteria.

(A) Univariate and multivariate analyses for PFS						
Covariates	Crude HR	95% CI	*p*	Adjusted HR	95% CI	*p*
AWGC cachexia vs. no cachexia	1.438	1.146–1.806	0.002	1.253	0.980–1.602	0.072
Age (≥ 75 vs. < 75 years)	1.061	0.827–1.362	0.640	1.061	0.811–1.389	0.667
ECOG PS (PS 0–1 vs. PS ≥ 2)	0.403	0.297–0.546	< 0.001	0.461	0.332–0.639	< 0.001
Histology (non‐sq vs. sq)	0.815	0.637–1.042	0.103	0.857	0.663–1.108	0.239
Stage (recurrence vs. IV)	0.766	0.569–1.030	0.077	0.776	0.571–1.054	0.104
PD‐L1 (90%–100% vs. 50%–89%)	0.640	0.503–0.814	< 0.001	0.600	0.468–0.768	< 0.001
Brain metastasis (yes vs. no) Liver	1.425	1.070–1.898	0.015	1.493	1.105–2.017	0.009
metastasis (yes vs. no)	1.391	1.011–1.915	0.043	1.222	0.870–1.716	0.248
Chemoimmunotherapy vs. ICI monotherapy	0.882	0.697–1.115	0.292	0.914	0.710–1.176	0.483

(B) Univariate and multivariate analyses for OS						
Covariates	Crude HR	95% CI	*p*	Adjusted HR	95% CI	*p*
AWGC cachexia vs. no cachexia	1.907	1.463–2.485	< 0.001	1.539	1.156–2.048	0.003
Age (≥ 75 vs. < 75 years)	1.572	1.188–2.081	0.002	1.426	1.048–1.941	0.024
ECOG PS (PS 0–1 vs. PS ≥ 2)	0.281	0.203–0.390	< 0.001	0.390	0.272–0.558	< 0.001
Histology (non‐sq vs. sq)	0.582	0.441–0.766	< 0.001	0.622	0.465–0.831	0.001
Stage (recurrence vs. IV)	0.543	0.365–0.807	0.003	0.559	0.372–0.841	0.005
PD‐L1 (90%–100% vs. 50%–89%)	0.768	0.581–1.014	0.063	0.694	0.521–0.924	0.012
Brain metastasis (yes vs. no) liver	1.315	0.945–1.830	0.104	1.551	1.098–2.190	0.013
metastasis (yes vs. no)	1.470	1.024–2.110	0.037	1.176	0.799–1.730	0.412
Chemoimmunotherapy vs. ICI monotherapy	0.758	0.569–1.010	0.058	0.871	0.640–1.184	0.377

Abbreviations: AWGC, Asian Working Group for Cachexia; CI, Confidence interval; ECOG PS, Eastern Cooperative Oncology Group performance status; HR, Hazard ratio; ICI, Immune checkpoint inhibitor; OS, Overall survival; PD‐L1, programmed cell death ligand 1; PFS, progression‐free survival; Sq, squamous cell carcinoma.

In the subset excluding patients with driver mutations (*n* = 390), AWGC‐defined cachexia remained significantly associated with shorter PFS and OS (median PFS: 7.2 vs. 13.4 months, *p* = 0.0008; median OS: 17.8 vs. 48.5 months, *p* < 0.0001) (Figure S1). In the multivariate Cox regression analysis, cachexia was also independently associated with worse OS (adjusted HR: 1.605, 95% CI: [1.196–2.153], *p* = 0.002) and PFS (adjusted HR: 1.317, 95% CI: [1.022–1.696], *p* = 0.033) (Table S2).

Similarly, Fearon‐defined cachexia was associated with significantly shorter OS (median OS: 18.4 vs. 37.7 months, *p* = 0.001), while the difference in PFS was not statistically significant, although a similar trend was observed (median PFS: 5.9 vs. 11.4 months, *p* = 0.06) (Figure 2c,d). In the multivariate analysis, Fearon‐defined cachexia remained significantly associated with worse OS (adjusted HR: 1.743, 95% CI: [1.226–2.479], *p* = 0.002) but not with PFS (adjusted HR: 1.326, 95% CI: [0.966–1.822], *p* = 0.081) (Table 3). We further assessed the effect of the treatment regimen in this population; however, no significant differences in therapeutic efficacy were observed between PD‐1/PD‐L1 inhibitor monotherapy and chemoimmunotherapy (Table 3).

**TABLE 3:** Univariate and multivariate analyses of PFS and OS according to Fearon cachexia criteria.

(A) Univariate and multivariate analyses for PFS						
Covariates	Crude HR	95% CI	*p*	Adjusted HR	95% CI	*p*
Fearon cachexia vs. no cachexia	1.340	0.982–1.830	0.065	1.326	0.966–1.822	0.081
Age (≥ 75 vs. < 75 years)	1.061	0.827–1.362	0.640	1.045	0.799–1.366	0.748
ECOG PS (PS 0–1 vs. PS ≥ 2)	0.403	0.297–0.546	< 0.001	0.450	0.326–0.623	< 0.001
Histology (non‐sq vs. sq)	0.815	0.637–1.042	0.103	0.821	0.635–1.062	0.134
Stage (recurrence vs. IV)	0.766	0.569–1.030	0.077	0.742	0.547–1.005	0.054
PD‐L1 (90%–100% vs. 50%–89%)	0.640	0.503–0.814	< 0.001	0.596	0.466–0.764	< 0.001
Brain metastasis (yes vs. no) liver	1.425	1.070–1.898	0.015	1.453	1.077–1.961	0.014
metastasis (yes vs. no)	1.391	1.011–1.915	0.043	1.339	0.961–1.866	0.085
Chemoimmunotherapy vs. ICI monotherapy	0.882	0.697–1.115	0.292	0.909	0.707–1.169	0.458

(B) Univariate and multivariate analyses for OS						
Covariates	Crude HR	95% CI	*p*	Adjusted HR	95% CI	*p*
Fearon cachexia vs. no cachexia	1.745	1.238–2.459	0.001	1.743	1.226–2.479	0.002
Age (≥ 75 vs. < 75 years)	1.572	1.188–2.081	0.002	1.368	1.007–1.857	0.045
ECOG PS (PS 0–1 vs. PS ≥ 2)	0.281	0.203–0.390	< 0.001	0.373	0.261–0.532	< 0.001
Histology (non‐sq vs. sq)	0.582	0.441–0.766	< 0.001	0.569	0.425–0.761	< 0.001
Stage (recurrence vs. IV)	0.543	0.365–0.807	0.003	0.501	0.333–0.752	< 0.001
PD‐L1 (90%–100% vs. 50%–89%)	0.768	0.581–1.014	0.063	0.699	0.525–0.931	0.014
Brain metastasis (yes vs. no) liver	1.315	0.945–1.830	0.104	1.452	1.029–2.048	0.034
metastasis (yes vs. no)	1.470	1.024–2.110	0.037	1.369	0.938–1.999	0.104
Chemoimmunotherapy vs. ICI monotherapy	0.758	0.569–1.010	0.058	0.841	0.619–1.141	0.266

Abbreviations: AWGC, Asian Working Group for Cachexia; CI, confidence interval; ECOG PS, Eastern Cooperative Oncology Group performance status; HR, hazard ratio; ICI, immune checkpoint inhibitor; OS, overall survival; PD‐L1, programmed cell death ligand 1; PFS, progression‐free survival; Sq, Squamous cell carcinoma.

In the subset excluding patients with driver mutations, Fearon‐defined cachexia was also significantly associated with shorter survival (median PFS: 5.2 vs. 11.4 months, *p* = 0.0066; median OS: 15.2 vs. 37.7 months, *p* < 0.001) (Figure S1). Multivariate analysis confirmed the independent association with OS (adjusted HR: 1.999, 95% CI: [1.391–2.874], *p* < 0.001) and PFS (adjusted HR: 1.544, 95% CI: [1.110–2.146], *p* = 0.009) (Table S3).

### 3.5 Comparison of Clinical Characteristics Among the Three Groups

To further investigate the clinical significance of cachexia according to these two definitions, patients were categorized into the following three groups (Figure 1b):

- Non cachexia group (*n* = 230): meeting neither the AWGC nor the Fearon criteria.
- The AWGC‐only cachexia group (A‐only cachexia) (*n* = 119) met only the AWGC criteria.
- The AWGC+Fearon cachexia group (A+F cachexia) (*n* = 49) met both the AWGC and Fearon criteria.

#### 3.5.1 Comparison Between the Noncachexia and AWGC‐Only Cachexia Groups

Compared to the noncachexia group, patients in the A‐only cachexia group had significantly lower BMI (median 20.55 vs. 23.01 kg/m^2^, *p* < 0.001), greater weight loss (1.0% vs. 0.0%, *p* < 0.001) and higher CRP levels (2.81 vs. 0.46 mg/dL, *p* < 0.001). ECOG PS ≥ 2 was more frequent in the A‐only cachexia group (16.0% vs. 8.7%, *p* = 0.049), as were liver metastases (23.5% vs. 9.1%, *p* = 0.001). In contrast, brain metastases were less common (10.1% vs. 19.1%; *p* = 0.031). There were also significant differences in the tumour characteristics. The A‐only cachexia group had a higher proportion of squamous cell carcinomas (37.8% vs. 24.8%, *p* = 0.021) and more frequently presented with Stage IV disease at the time of treatment initiation (90.8% vs. 75.7%, *p* = 0.001) (Table S4).

#### 3.5.2 Comparison Between the AWGC‐Only Cachexia and AWGC+Fearon Cachexia Groups

Compared to the A‐only cachexia group, the A+F cachexia group had significantly lower BMI (median 17.81 vs. 20.55 kg/m^2^, *p* < 0.001), greater weight loss (8.0% vs. 1.0%, *p* < 0.001), higher CRP levels (4.7 vs. 2.8 mg/dL, *p* = 0.017) and higher frequency of ECOG PS ≥ 2 (34.7% vs. 16.0%, *p* = 0.012). The metastatic distribution was comparable, including liver (23.5% vs. 14.3%, *p* = 0.214) and brain metastases (10.1% vs. 14.3%, *p* = 0.432). There were no significant differences in the histology or frequency of Stage IV disease at treatment initiation (Table S5).

Furthermore, categorical comparisons of cachexia‐defining variables revealed that no patients in the A+F cachexia group had a BMI ≥ 20 kg/m^2^, whereas nearly 70% of patients in the A‐only group were within this range. Over 70% of patients in the A+F cachexia group experienced ≥ 5% weight loss, compared to < 25% in the A‐only group. All patients in both groups had CRP levels ≥ 0.5 mg/dL. These findings underscore the more severe metabolic and inflammatory state of the A+F cachexia group, even within the AWGC‐defined cachexia category (Table S6).

#### 3.5.3 Nutritional and Inflammatory Markers Across the Three Groups

We compared the cachexia‐related indices across the three groups and observed a stepwise gradient from the noncachexia group to the A‐only cachexia group and then to the A+F cachexia group. BMI (noncachexia / A‐only cachexia / A+F cachexia: 23.01 [15.28–35.12] / 20.55 [13.97–26.91] / 17.81 [14.44–19.93] kg/m^2^, *p* < 0.001), weight loss (noncachexia / A‐only cachexia / A+F cachexia: 0.0% [−9.0%, 27.0%] /1.0% [−3.0%, 22.0%] / 8.0% [2.0%, 23.0%], *p* < 0.001), CRP (noncachexia / A‐only cachexia / A+F cachexia: 0.46 [0.00–26.00] /2.81 [0.52–21.65] / 4.74 [0.55–19.44] mg/dL, *p* < 0.001), the neutrophil‐to‐lymphocyte ratio (noncachexia / A‐only cachexia / A+F cachexia: 3.53 [0.60–79.71] /5.08 [1.12–89.00] / 7.25 [1.23–54.88], *p* < 0.001), the prognostic nutritional index (noncachexia / A‐only cachexia / A+F cachexia: 44.53 [20.45–67.31] /40.11 [18.99–60.98] /34.91 [17.31–58.20], *p* < 0.001) and geriatric nutritional risk index (noncachexia / A‐only cachexia/A+F cachexia: 99.24 [63.85–137.71] /88.14 [56.58–112.15] /78.40 [53.08–95.84], *p* < 0.001) showed concordant differences across cohorts. In each case, the A‐only cachexia group was intermediate between the noncachexia and A+F cachexia groups (Figure S2).

### 3.6 Comparison of Clinical Outcomes Among the Three Groups

#### 3.6.1 Comparison Between the Noncachexia and AWGC‐Only Cachexia Groups

**FIGURE 3:**
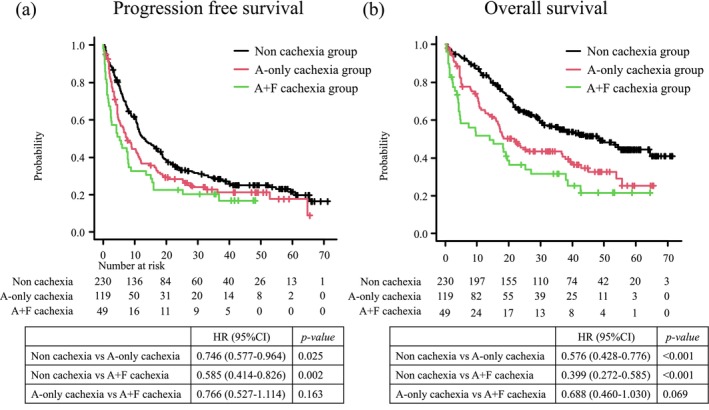
Kaplan–Meier survival curves among three groups based on the Asian Working Group for Cachexia (AWGC) and Fearon criteria. Patients were categorized into the noncachexia, A‐only cachexia (meeting only the AWGC criteria) and A+F cachexia (meeting both the AWGC and Fearon criteria) groups. (a) Progression‐free survival across the three groups. (b) Overall survival across the three groups.

Median PFS was significantly shorter in the A‐only cachexia group than in the noncachexia group (7.46 vs. 13.08 months, *p* = 0.025), as was median OS (21.84 vs. 48.49 months, *p* = 0.0002) (Figure 3a,b). In the multivariate Cox regression analysis adjusted for age, sex, ECOG PS, histology, stage, PD‐L1 status, presence of metastases and treatment regimen, OS remained significantly longer in the noncachexia group (adjusted HR, 0.699; 95% CI: [0.505–0.969], *p* = 0.031), whereas the difference in PFS showed a nonsignificant trend (adjusted HR, 0.833; 95% CI: [0.630–1.102]; *p* = 0.200) (Table 4).

**TABLE 4:** Univariate and multivariate analyses of survival outcomes between the non‐cachexia and AWGC‐only cachexia groups.

(A) Univariate and multivariate analyses for PFS						
Covariates	Crude HR	95% CI	*p*	Adjusted HR	95% CI	*p*
Non‐cachexia vs. A‐only cachexia	0.746	0.577–0.964	0.025	0.833	0.630–1.102	0.200
Age (≥ 75 vs. < 75 years)	1.060	0.808–1.389	0.675	1.087	0.812–1.456	0.575
ECOG PS (PS 0–1 vs. PS ≥ 2)	0.438	0.305–0.628	< 0.001	0.490	0.335–0.717	< 0.001
Histology (non‐sq vs. sq)	0.825	0.632–1.076	0.155	0.883	0.667–1.167	0.381
Stage (recurrence vs. IV)	0.762	0.554–1.049	0.095	0.779	0.560–1.084	0.139
PD‐L1 (90%–100% vs. 50%–89%)	0.634	0.487–0.825	< 0.001	0.583	0.444–0.765	< 0.001
Brain metastasis (yes vs. no) liver	1.412	1.034–1.929	0.030	1.501	1.080–2.084	0.016
metastasis (yes vs. no)	1.356	0.961–1.914	0.083	1.243	0.860–1.796	0.247
Chemoimmunotherapy vs. ICI monotherapy	0.929	0.720–1.200	0.574	0.905	0.687–1.192	0.477

(B) Univariate and multivariate analyses for OS						
Covariates	Crude HR	95% CI	*p*	Adjusted HR	95% CI	*p*
Non‐cachexia vs. A‐only cachexia	0.576	0.428–0.776	< 0.001	0.699	0.505–0.969	0.031
Age (≥ 75 vs. < 75 years)	1.634	1.201–2.223	0.001	1.567	1.117–2.198	0.009
ECOG PS (PS 0–1 vs. PS ≥ 2)	0.320	0.217–0.473	< 0.001	0.448	0.294–0.684	< 0.001
Histology (non‐sq vs. sq)	0.563	0.417–0.761	< 0.001	0.618	0.449–0.850	0.003
Stage (recurrence vs. IV)	0.471	0.299–0.742	0.001	0.510	0.319–0.816	0.005
PD‐L1 (90%–100% vs. 50%–89%)	0.765	0.561–1.043	0.090	0.665	0.484–0.914	0.012
Brain metastasis (yes vs. no) liver	1.321	0.917–1.903	0.135	1.645	1.120–2.417	0.011
metastasis (yes vs. no)	1.433	0.963–2.132	0.076	1.253	0.821–1.915	0.296
Chemoimmunotherapy vs. ICI monotherapy	0.776	0.564–1.068	0.120	0.857	0.608–1.206	0.375

Abbreviations: A‐only cachexia, AWGC‐only cachexia; AWGC, Asian Working Group for Cachexia; CI, confidence interval; ECOG PS, Eastern Cooperative Oncology Group performance status; HR, hazard ratio; ICI, immune checkpoint inhibitor; OS, overall survival; PD‐L1, programmed cell death ligand 1; PFS, Progression free survival; Sq, squamous cell carcinoma.

#### 3.6.2 Comparison Between the AWGC‐Only Cachexia and AWGC+Fearon Cachexia Groups

The median PFS was shorter in the A+F cachexia group than in the A‐only cachexia group (5.22 vs. 7.46 months, *p* = 0.163). Median OS was also shorter (13.93 vs. 21.85 months, *p* = 0.069) (Figure 3a,b). In the multivariate Cox regression analysis adjusted for age, sex, ECOG PS, histology, stage, PD‐L1 status, presence of metastases and treatment regimen, the differences did not reach statistical significance; however, both PFS and OS were better in the A‐only cachexia group (PFS: adjusted HR 0.728, 95% CI: [0.491–1.079], *p* = 0.114; OS: adjusted HR 0.674, 95% CI: [0.441–1.031], *p* = 0.068) (Table 5).

**TABLE 5:** Univariate and multivariate analyses of survival outcomes between the AWGC‐only cachexia and AWGC+Fearon cachexia groups.

(A) Univariate and multivariate analyses for PFS						
Covariates	Crude HR	95% CI	*p*	Adjusted HR	95% CI	*p*
A‐only cachexia vs. A+F cachexia	0.766	0.527–1.114	0.163	0.728	0.491–1.079	0.114
Age (≥ 75 vs. < 75 years)	1.226	0.834–1.803	0.300	1.042	0.675–1.608	0.853
ECOG PS (PS 0–1 vs. PS ≥ 2)	0.542	0.361–0.813	0.003	0.605	0.383–0.956	0.031
Histology (non‐sq vs. sq)	0.747	0.523–1.067	0.109	0.710	0.483–1.043	0.080
Stage (recurrence vs. IV)	0.842	0.474–1.494	0.556	0.714	0.392–1.301	0.271
PD‐L1 (90%–100% vs. 50%–89%)	0.763	0.534–1.091	0.138	0.779	0.535–1.133	0.192
Brain metastasis (yes vs. no) liver	1.241	0.732–2.103	0.422	1.163	0.674–2.005	0.587
metastasis (yes vs. no)	1.239	0.819–1.874	0.310	1.315	0.835–2.072	0.237
Chemoimmunotherapy vs. ICI monotherapy	0.774	0.541–1.108	0.162	0.873	0.582–1.311	0.514

(B) Univariate and multivariate analyses for OS						
Covariates	Crude HR	95% CI	*p*	Adjusted HR	95% CI	*p*
A‐only cachexia vs. A+F cachexia	0.688	0.460–1.030	0.069	0.674	0.441–1.031	0.068
Age (≥ 75 vs. < 75 years)	1.629	1.083–2.451	0.019	1.340	0.837–2.146	0.223
ECOG PS (PS 0–1 vs. PS ≥ 2)	0.422	0.276–0.644	< 0.001	0.480	0.297–0.774	0.003
Histology (non‐sq vs. sq)	0.681	0.462–1.002	0.051	0.633	0.418–0.959	0.031
Stage (recurrence vs. IV)	0.896	0.480–1.672	0.731	0.710	0.367–1.374	0.310
PD‐L1 (90%–100% vs. 50%–89%)	0.701	0.472–1.042	0.079	0.717	0.474–1.083	0.114
Brain metastasis (yes vs. no) liver	1.281	0.742–2.211	0.374	1.216	0.686–2.158	0.503
metastasis (yes vs. no)	1.224	0.778–1.928	0.382	1.169	0.708–1.932	0.542
Chemoimmunotherapy vs. ICI monotherapy	0.785	0.526–1.173	0.237	0.998	0.635–1.568	0.993

Abbreviations: A‐only cachexia, AWGC‐only cachexia; A+F cachexia, AWGC+Fearon cachexia; AWGC, Asian Working Group for Cachexia; CI, confidence interval; ECOG PS, Eastern Cooperative Oncology Group performance status; HR, hazard ratio; ICI, immune checkpoint inhibitor; OS, overall survival; PD‐L1, programmed cell death ligand 1; PFS, progression free survival; Sq, squamous cell carcinoma.

Taken together, the clinical outcomes in patients with NSCLC receiving systemic therapy, including immunotherapy, differed across the three groups in the following order: no‐cachexia group > AWGC‐only cachexia group > AWGC + Fearon cachexia group, with the AWGC + Fearon cachexia group exhibiting the poorest outcomes.

## 4 Discussion

In this study, the AWGC and Fearon criteria demonstrated comparable utility as predictors of first‐line immunotherapy efficacy and prognosis. Importantly, the subgroup classified as having cachexia solely according to the AWGC criteria (A‐only cachexia group) represented a distinct clinical population different from both the noncachexia group and the classical patient population defined by the Fearon criteria. Although this subgroup exhibited a response to immunotherapy similar to that of the noncachexia group, the survival was significantly shorter. Furthermore, their clinical outcomes differed from those of the patients who met both the AWGC and Fearon criteria.

The AWGC framework was specifically developed to account for Asian body size and composition, which are not adequately reflected in the Fearon criteria [1, 8, 11, 12]. In this cohort, nearly one‐third of the patients were classified as having cachexia by the AWGC, including 119 individuals (29%) overlooked by the Fearon definition. Unexpectedly, the overlap between the two definitions was limited, with only 49 patients (11.9%) meeting both criteria; approximately 20% of Fearon‐positive cases (*n* = 13) were classified as noncachexia according to the AWGC criteria (Figure 1). These findings clearly demonstrate the important differences between the two criteria.

We also compared patient characteristics and outcomes according to the presence or absence of cachexia as defined by the AWGC and Fearon criteria. In both definitions, the cachexia groups showed unfavourable body composition and inflammatory profiles, whereas the AWGC criteria further identified differences in histology, stage and liver or brain metastases (Table 1). Regarding the outcomes, PFS did not differ significantly, but OS was consistently shorter in the cachexia groups (Tables 2 and 3). The HRs for PFS and OS were comparable, indicating that both criteria provided clinically meaningful information. Consistent with a large Chinese cohort study, which also found no substantial difference in HRs between the two definitions [13], these findings suggest that neither is inferior, and both are important for characterizing cachexia in Asian patients.

This study is the first to comprehensively characterize the clinical features and outcomes of the newly identified cachexia population as determined by the AWGC criteria. In this study, we showed that these patients, although previously classified as noncachexic, were less likely to have an ECOG PS of 0–1 and exhibited significant differences in weight loss, CRP levels, BMI, the prognostic nutritional and geriatric nutritional risk indices and markers of chronic inflammation (Figure S2). These results suggest that the A‐only cachexia group had a worse nutritional and inflammatory status than the noncachexia group but showed milder severity than the A+F cachexia group (Figure S2) [1].

We examined which cachexia‐related components accounted for the distinction between the A‐only and A+F groups among patients classified according to the AWGC criteria (Table S6). The key difference was that more than half (58.8%) of patients in the A‐only group had weight loss of < 2%. According to the AWGC definition, patients with a BMI < 21 kg/m^2^ (the cutoff utilized in the AWGC criteria) are classified as having cachexia regardless of weight loss if CRP level is elevated; thus, this group was predominantly characterized by “absolute low BMI (< 21 kg/m^2^) with minimal weight loss (< 2%).” This profile fundamentally differs from the Fearon definition (typically requiring BMI < 20 kg/m^2^ combined with weight loss), which posits ongoing weight loss as the central driver of diagnosis [8]. In Western populations, where the baseline BMI is significantly higher, patients reaching a BMI of < 21 kg/m^2^ have typically already undergone profound weight loss, thereby meeting the Fearon criteria as well. Therefore, the “A‐only” phenotype—characterized by low absolute muscle/fat mass with systemic inflammation but without severe weight loss—appears to be a clinical entity specific to the Asian population, likely reflecting a “constitutionally lean” baseline. Applying these criteria to Western populations may be less relevant, as cachexia in those cohorts is almost exclusively driven by the trajectory of weight loss rather than static low BMI. Collectively, our findings suggest that the AWGC criteria are optimized for capturing this Asian‐specific “early/lean” cachexia, and caution is warranted when generalizing these findings to non‐Asian populations.

Experimental and translational studies have demonstrated that inflammation is evident in mild stage cachexia, even before substantial weight loss becomes apparent. Tumour‐ and host‐derived cytokines, particularly interleukin (IL)‐6, IL‐1 and tumour necrosis factor‐alpha, are upregulated early and contribute to anorexia through hypothalamic signalling while simultaneously accelerating skeletal muscle wasting via activation of the ubiquitin–proteasome and autophagy–lysosome pathways [2, 14, 15, 16, 17]. These pro‐inflammatory signals also trigger hepatic acute‐phase responses, with CRP frequently elevated, even in early‐stage patients [18]. Taken together, these findings indicate that the mild stage cachexia is biologically active, characterized by systemic inflammation and metabolic derangements, and should be recognized as a critical window for early intervention, rather than a clinically silent stage [14, 16]. These early inflammatory and metabolic alterations are also known to influence the host immune environment, thereby potentially affecting the efficacy of immune checkpoint inhibitors.

From the perspective of immunotherapy, early intervention may have important clinical implications. Cachexia has been associated with attenuated efficacy of immunotherapy, likely mediated by systemic inflammation and metabolic dysregulation [5, 19]. Basic research utilizing tumour‐bearing mouse models has demonstrated that neutralizing inflammatory cytokines, such as IL‐6, at an early stage can effectively reverse metabolic dysfunction and muscle wasting, whereas late‐stage intervention often fails to rescue the phenotype [20]. This highlights the existence of a window of opportunity for intervention. Conversely, recent studies have suggested that patients whose cachexia improves during treatment may achieve better responses to immunotherapy and prolonged survival [21]. However, such improvement has been observed in only a small subset of patients, generally restricted to those with minimal weight loss, typically less than approximately 3% over 6 months [22]. In this context, the newly identified A‐only group in the present study may represent a clinically relevant population for early intervention. This group exhibited a median weight loss of approximately 1% over 6 months and relatively low levels of systemic inflammation, suggesting that this subgroup may reflect a potentially reversible or distinct stage of cachexia. Early identification and intervention in this population may therefore help preserve immunotherapy efficacy before progression to more advanced cachexia.

Considering these findings, the group newly identified by the AWGC criteria (the A‐only cachexia group) appears to represent mild stage cachexia, as these patients had < 5% weight loss but higher inflammation than did those in the noncachexia group. Despite showing comparable efficacy to first‐line treatment, the OS was significantly shorter, indicating that patients in this category may already be on a trajectory toward poorer outcomes. Recent clinically applied cachexia‐targeted therapeutic strategies should also be considered for early intervention. In fact, anamorelin, a ghrelin receptor agonist, has shown benefits in patients with preserved PS, with diminished effects once the functional decline has advanced [23, 24, 25, 26]. Reports on gastrointestinal cancers suggest that early introduction may prolong survival [27]. We are conducting a prospective observational study (SPIRAL‐ANA study; JRCT 1071210053) to evaluate the safety and efficacy of adding anamorelin to first‐line chemoimmunotherapy in patients with NSCLC, with the expectation that early anticachexia intervention may improve treatment outcomes.

This study had some limitations. First, the retrospective design of the study may have introduced an unintended bias. Second, as the study population was limited to approximately 400 Japanese patients, the extent to which these findings can be extrapolated to other Asian populations remains unclear. However, comparable data have been documented in Chinese cohorts, suggesting the potential for extrapolation [13]. Third, owing to the retrospective study design, we could not assess handgrip strength, which is one of the AWGC criteria, and the actual prevalence of cachexia might therefore be higher than previously reported. Similarly, the Fearon criteria require assessment of sarcopenia, which could not be performed in this study. Consequently, some patients with sarcopenia may not have been identified and may have been misclassified as noncachectic, potentially influencing the observed prognostic differences between groups. This potential misclassification could dilute the survival difference between the noncachexia and cachexia groups, thereby underestimating the prognostic impact of cachexia.

## 5 Conclusion

In NSCLC patients treated with immunotherapy, the Asian Working Group for Cachexia criteria demonstrated prognostic utility comparable to that of the Fearon definition, while additionally identifying a distinct subgroup corresponding to “Hidden cachexia.” Although this subgroup maintained treatment response to immunotherapy, OS was significantly shorter, suggesting that early identification of such patients may aid risk stratification and inform future therapeutic strategies.

## Funding

The authors have nothing to report.

## Ethics Statement

The study protocol was approved by the ethics committee of the Kyoto Prefectural University of Medicine and received additional approval from the institutional review boards of the 13 participating hospitals (approval no. ERB‐C‐2113).

## Conflicts of Interest

N. Nishioka has received personal fees from Chugai Pharmaceutical Co. Ltd., AstraZeneca KK., Eli Lilly Japan KK and MSD KK outside the purview of the submitted work. H. Kawachi has received personal fees from Amgen, AstraZeneca, Bristol‐Myers Squibb, Chugai Pharmaceutical, Eli Lilly Japan, Janssen, MSD, Ono Pharmaceutical and Taiho Pharmaceutical outside the purview of the submitted work. T. Yamada has received research grants from TAIYO CHEMICAL INDUSTRY Co. Ltd. and has received speaking honoraria from Eli Lilly and Chugai Pharmaceutical outside the purview of the submitted work. M. Tamiya has received research grants from Boehringer Ingelheim, Ono Pharmaceutical, Bristol‐Myers Squibb, MSD, Daiichi‐Sankyo, Eisai, Chugai Pharmaceutical and Janssen and personal fees from Chugai Pharmaceutical, Boehringer Ingelheim, AstraZeneca, Taiho Pharmaceutical, Eli Lilly, Novartis, Pfizer, Asahi Kasei Pharmaceutical, Ono Pharmaceutical, Bristol‐Myers Squibb, MSD, Bayer, Amgen, Kyowa‐Kirin and Nippon Kayaku, all outside the purview of the submitted work. A. Okada has received personal fees from Chugai‐Roshe, AstraZeneca, Boehringer Ingelheim, Eli Lilly, Japan, Nippon Kayaku and Bristol Myers Squibb outside the purview of the submitted work. T. Kijima has received personal fees from Chugai Pharmaceutical Co. Ltd. and MSD KK outside the purview of the submitted work. K. Takayama has received research grants from Chugai Pharmaceutical and Ono Pharmaceutical and personal fees from AstraZeneca, Chugai Pharmaceutical, MSD‐Merck, Eli Lilly, Boehringer‐Ingelheim and Daiichi‐Sankyo outside the purview of the submitted work. The other authors declare no potential conflicts of interest.

## Supporting information


**Figure S1:** Kaplan–Meier survival curves in patients excluding driver mutations, comparing the cachexia and noncachexia groups defined by the Asian Working Group for Cachexia (AWGC) and Fearon criteria. (a) Progression‐free survival (PFS) according to AWGC‐defined cachexia status. (b) Overall survival (OS) according to AWGC‐defined cachexia status. (c) PFS according to Fearon‐defined cachexia status. (d) OS according to Fearon‐defined cachexia status.

**Figure S2:** Nutritional and inflammatory markers across three groups. Patients were classified into three groups: noncachexia, A‐only cachexia (meeting only the Asian Working Group for Cachexia [AWGC] criteria) and A+F cachexia (meeting both the AWGC and Fearon criteria) groups. We compared (a) body weight loss, (b) body mass index (BMI), (c) C‐reactive protein (CRP), (d) neutrophil‐to‐lymphocyte ratio (NLR), (e) prognostic nutritional index (PNI) and (f) geriatric nutritional risk index (GNRI) across these three groups. NLR was calculated as neutrophil count divided by lymphocyte count. PNI was calculated as 10 × serum albumin (g/dL) + 0.005 × total lymphocyte count (/μL). GNRI was calculated as [1.489 × serum albumin (g/L)] + [41.7 × (current body weight/ideal body weight)].

**Table S1:** Patient characteristics in the overall population.

**Table S2:** Univariate and multivariate analyses of PFS and OS according to AWGC cachexia criteria in patients without driver mutations.

**Table S3:** Univariate and multivariate analyses of PFS and OS according to Fearon's cachexia criteria in patients without driver mutations.

**Table S4:** Patients' characteristics (no‐cachexia vs. A‐only cachexia).

**Table S5:** Patients' characteristics (A‐only cachexia vs. A+F cachexia).

**Table S6:** Comparison of diagnostic criteria components between A‐only cachexia vs. A+F cachexia.
