# Supplementary material for: Identification of Hidden Cachexia Subgroup in PD‐L1‐High NSCLC: Comparative Analysis of the AWGC vs. Fearon Criteria
Source: J Cachexia Sarcopenia Muscle. 2026 Apr 12;17(2):e70281. doi: 10.1002/jcsm.70281 (PMC13070542; doi:10.1002/jcsm.70281)
Supplement: Supplementary file 1 — Figure S1: Kaplan–Meier survival curves in patients excluding driver mutations, comparing the cachexia and noncachexia groups defined by the Asian Working Group for Cachexia (AWGC) and Fearon criteria. (a) Progression‐free survival (PFS) according to AWGC‐defined cachexia status. (b) Overall survival (OS) according to AWGC‐defined cachexia status. (c) PFS according to Fearon‐defined cachexia status. (d) OS according to Fearon‐defined cachexia status. [file JCSM-17-e70281-s005.pptx]

## Slide 1
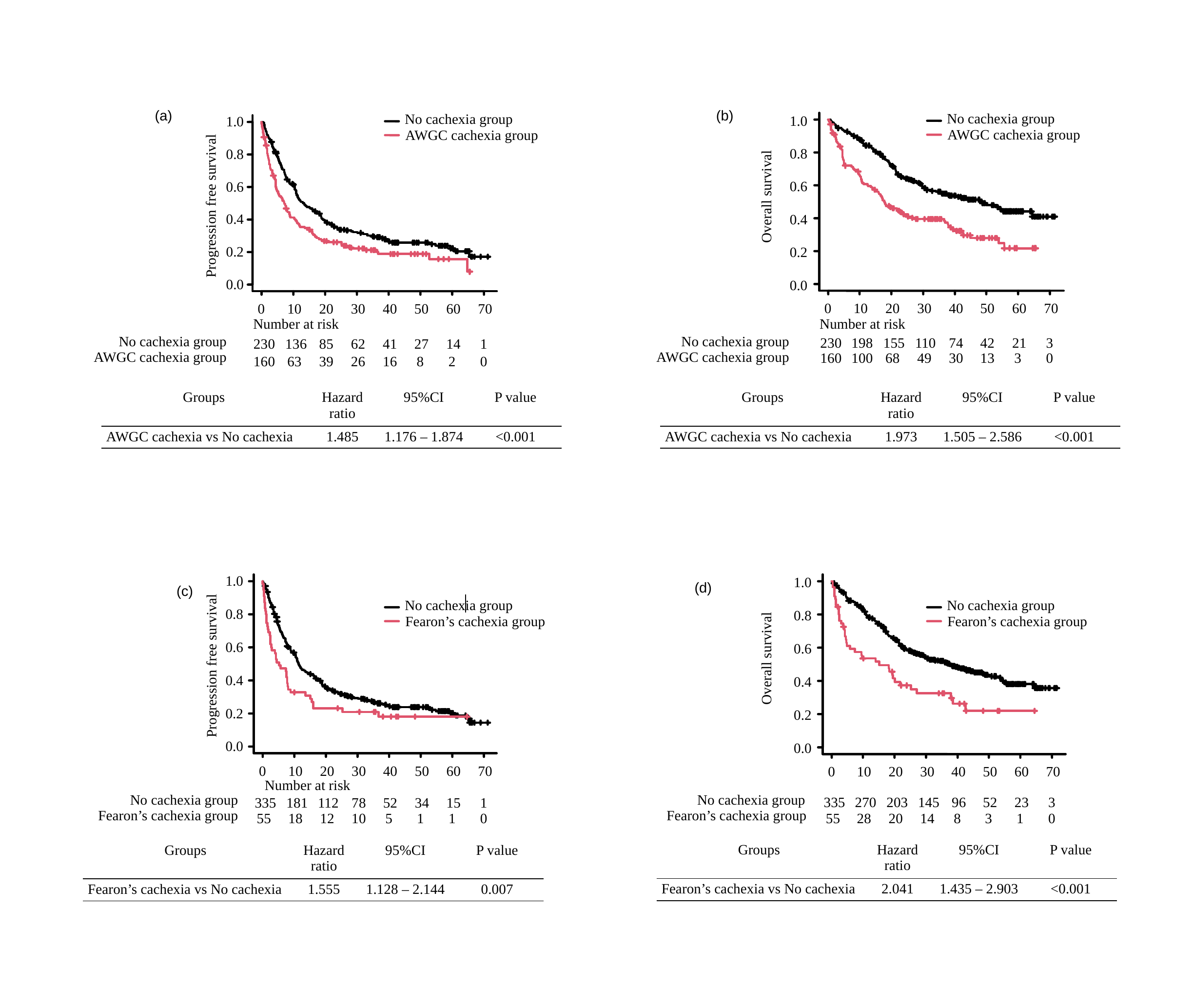

(a)
(b)
No cachexia group
No cachexia group
1.0
0.8
0.6
0.4
0.2
0.0
0
10
20
30
40
50
60
70
230
198
155
110
74
42
21
3
160
100
68
49
30
13
3
0
1.0
0.8
0.6
0.4
0.2
0.0
0
10
20
30
40
50
60
70
230
136
85
62
41
27
14
1
160
63
39
26
16
8
2
0
AWGC cachexia group
AWGC cachexia group
Overall survival
Progression free survival
Number at risk
Number at risk
No cachexia group
No cachexia group
AWGC cachexia group
AWGC cachexia group
| Groups | Hazard ratio | 95%CI | P value |
| --- | --- | --- | --- |
| AWGC cachexia vs No cachexia | 1.485 | 1.176 – 1.874 | <0.001 |
| Groups | Hazard ratio | 95%CI | P value |
| --- | --- | --- | --- |
| AWGC cachexia vs No cachexia | 1.973 | 1.505 – 2.586 | <0.001 |
1.0
0.8
0.6
0.4
0.2
0.0
0
10
20
30
40
50
60
70
335
181
112
78
52
34
15
1
55
18
12
10
5
1
1
0
1.0
0.8
0.6
0.4
0.2
0.0
0
10
20
30
40
50
60
70
335
270
203
145
96
52
23
3
55
28
20
14
8
3
1
0
(d)
(c)
No cachexia group
No cachexia group
Fearon’s cachexia group
Fearon’s cachexia group
Overall survival
Progression free survival
Number at risk
No cachexia group
No cachexia group
Fearon’s cachexia group
Fearon’s cachexia group
| Groups | Hazard ratio | 95%CI | P value |
| --- | --- | --- | --- |
| Fearon’s cachexia vs No cachexia | 2.041 | 1.435 – 2.903 | <0.001 |
| Groups | Hazard ratio | 95%CI | P value |
| --- | --- | --- | --- |
| Fearon’s cachexia vs No cachexia | 1.555 | 1.128 – 2.144 | 0.007 |
