# Supplementary material for: Identification of Hidden Cachexia Subgroup in PD‐L1‐High NSCLC: Comparative Analysis of the AWGC vs. Fearon Criteria
Source: J Cachexia Sarcopenia Muscle. 2026 Apr 12;17(2):e70281. doi: 10.1002/jcsm.70281 (PMC13070542; doi:10.1002/jcsm.70281)
Supplement: Supplementary file 2 — Figure S2: Nutritional and inflammatory markers across three groups. Patients were classified into three groups: noncachexia, A‐only cachexia (meeting only the Asian Working Group for Cachexia [AWGC] criteria) and A+F cachexia (meeting both the AWGC and Fearon criteria) groups. We compared (a) body weight loss, (b) body mass index (BMI), (c) C‐reactive protein (CRP), (d) neutrophil‐to‐lymphocyte ratio (NLR), (e) prognostic nutritional index (PNI) and (f) geriatric nutritional risk index (GNRI) across these three groups. NLR was calculated as neutrophil count divided by lymphocyte count. PNI was calculated as 10 × serum albumin (g/dL) + 0.005 × total lymphocyte count (/μL). GNRI was calculated as [1.489 × serum albumin (g/L)] + [41.7 × (current body weight/ideal body weight)]. [file JCSM-17-e70281-s004.pptx]

## Slide 1
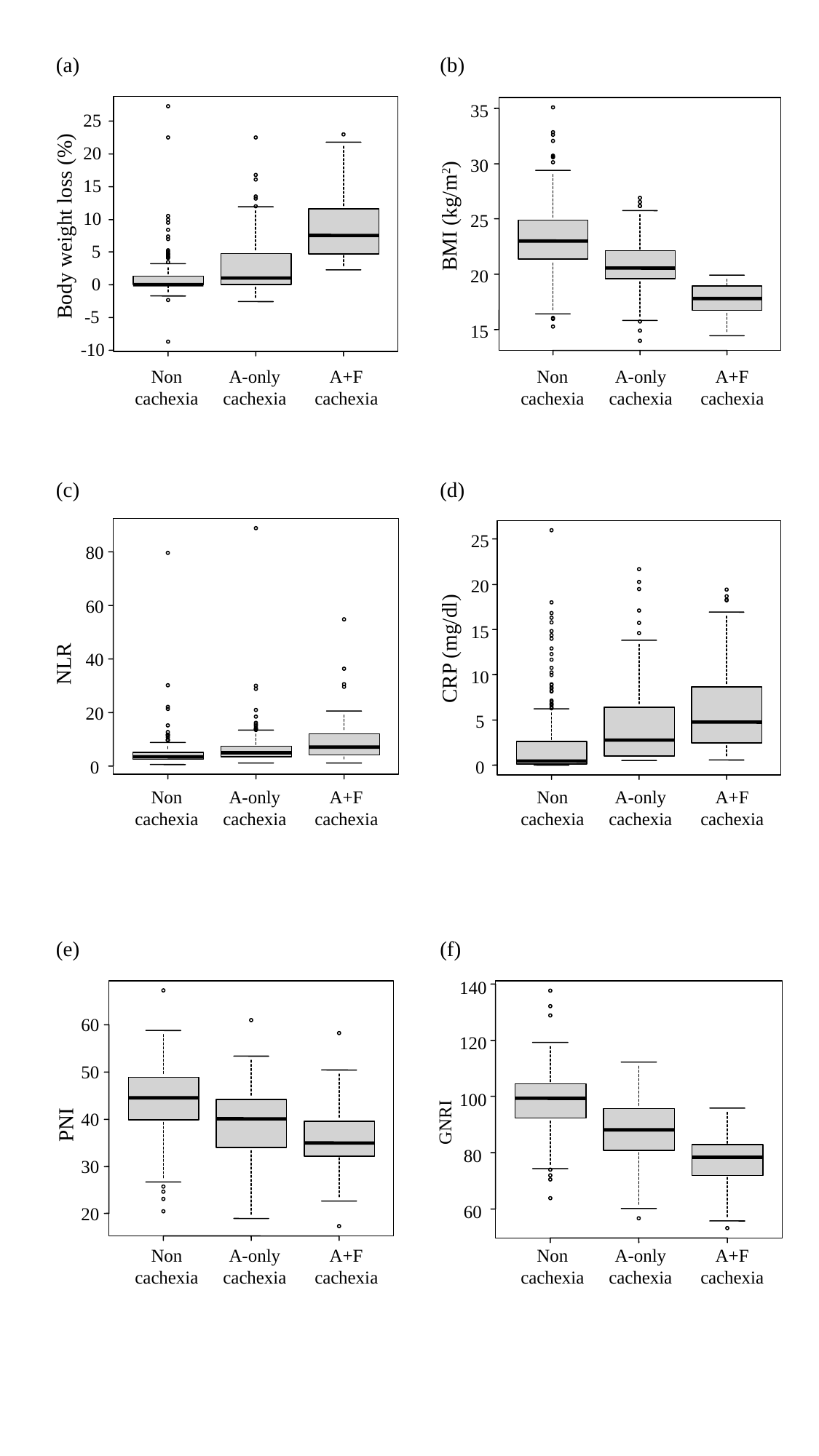

(a)
(b)
25
20
15
10
 5
 0
-5
-10
35
30
25
20
15
BMI (kg/m2)
Body weight loss (%)
Non
cachexia
A-only
cachexia
A+F
cachexia
Non
cachexia
A-only
cachexia
A+F
cachexia
(c)
(d)
80
60
40
20
0
25
20
15
10
5
0
CRP (mg/dl)
NLR
Non
cachexia
A-only
cachexia
A+F
cachexia
Non
cachexia
A-only
cachexia
A+F
cachexia
(e)
(f)
140
120
100
80
60
60
50
40
30
20
GNRI
PNI
Non
cachexia
A-only
cachexia
A+F
cachexia
Non
cachexia
A-only
cachexia
A+F
cachexia
