# Supplementary material for: Identification of Hidden Cachexia Subgroup in PD‐L1‐High NSCLC: Comparative Analysis of the AWGC vs. Fearon Criteria
Source: J Cachexia Sarcopenia Muscle. 2026 Apr 12;17(2):e70281. doi: 10.1002/jcsm.70281 (PMC13070542; doi:10.1002/jcsm.70281)
Supplement: Supplementary file 3 — Table S1: Patient characteristics in the overall population. [file JCSM-17-e70281-s003.docx]

**Supplementary Table1.Patient Characteristics in the Overall Population**

| **Characteristic** | **Total**  **(n=411)** |
| --- | --- |
|  |  |
| Age, y  Median (range) | 71 [36-90] |
| Sex  Male  Female | 320 (77.9)  91 (22.1) |
| ECOG PS  0-1  **≥** 2 | 354 (86.1)  57 (13.9) |
| BMI (kg/m^2^) | 21.4 [13.9-35.1] |
| Body weight loss (%) | 1.0 [-9.0-27.0] |
| CRP (mg/dl) | 1.4 [0.0-26.0] |
| Smoking history  Yes  No | 350 (85.2)  61 (14.8) |
| Stage  IV  Postoperative recurrence | 335 (81.5)  76 (18.5) |
| Histology  Squamous cell carcinoma  Adenocarcinoma  Others | 115 (28.0)  239 (58.2)  57 (13.8) |
| Driver gene alteration  EGFR  ALK  ROS1 | 14 (3.4)  6 (1.5)  1 (0.2) |
| Liver metastasis | 56 (13.6) |
| Brain metastasis | 67 (16.3) |
| Programmed cell death ligand 1, %  50-89  90-100 | 259 (63.0)  152 (37.0) |
| Anamorelin administration | 1 (0.2) |
| Treatment regimen  Pembrolizumab monotherapy  Chemoimmunotherapy | 255 (62.0)  156 (38.0) |

ECOG-PS, Eastern Cooperative Oncology Group performance status; BMI, Body mass index; EGFR, Epidermal Growth Factor Receptor; ALK, Anaplastic Lymphoma Kinase; ROS1, Receptor Oncogene Serine/threonine kinase 1
