# Supplementary material for: Identification of Hidden Cachexia Subgroup in PD‐L1‐High NSCLC: Comparative Analysis of the AWGC vs. Fearon Criteria
Source: J Cachexia Sarcopenia Muscle. 2026 Apr 12;17(2):e70281. doi: 10.1002/jcsm.70281 (PMC13070542; doi:10.1002/jcsm.70281)
Supplement: Supplementary file 4 — Table S2: Univariate and multivariate analyses of PFS and OS according to AWGC cachexia criteria in patients without driver mutations. [file JCSM-17-e70281-s002.docx]

**Supplementary Table 2. Univariate and Multivariate Analyses of PFS and OS According to AWGC Cachexia Criteria in Patients Without Driver Mutations**

| Covariates | Crude HR | 95% CI | p-value | Adjusted HR | 95% CI | p-value |
| --- | --- | --- | --- | --- | --- | --- |
| AWGC cachexia vs No cachexia  Age (≥75 vs <75) | 1.485  1.099 | 1.176 - 1.874  0.854 - 1.415 | <0.001  0.464 | 1.317  1.080 | 1.022 - 1.696  0.823 - 1.418 | 0.033  0.580 |
| ECOG-PS (PS 0-1 vs PS ≥2)  Histology (Non-Sq vs Sq) | 0.411  0.828 | 0.301 - 0.561  0.645 - 1.063 | <0.001  0.139 | 0.477  0.893 | 0.341 - 0.666  0.688 - 1.160 | <0.001  0.397 |
| Stage (Recurrence vs IV)  PD-L1 (90-100% vs 50%-89%) | 0.792  0.635 | 0.587 - 1.070  0.495 - 0.813 | 0.129  <0.001 | 0.790  0.583 | 0.578 - 1.079  0.452 - 0.754 | 0.138  <0.001 |
| Brain metastasis (Yes vs No)  liver metastasis (Yes vs No)  CI therapy vs ICI monotherapy | 1.303  1.385  0.877 | 0.958 - 1.772  0.996 - 1.928  0.689 - 1.117 | 0.092  0.053  0.287 | 1.392  1.211  0.889 | 1.008 - 1.922  0.853 - 1.717  0.684 – 1.155 | 0.044  0.284  0.378 |

1. Univariate and Multivariate analysis for PFS

PFS, Progression free survival; OS, Overall survival; HR, Hazard ratio; AWGC, Asian Working Group for Cachexia; ECOG-PS, Eastern Cooperative Oncology Group performance status; Sq, Squamous cell carcinoma; CI, ChemoImmunotherapy; ICI, immune checkpoint inhibitor; PD-L1, Programmed Death Ligand-1

1. Univariate and Multivariate analysis for OS

| Covariates | Crude HR | 95% CI | p-value | Adjusted HR | 95% CI | p-value |
| --- | --- | --- | --- | --- | --- | --- |
| AWGC cachexia vs No cachexia  Age (≥75 vs <75) | 1.973  1.611 | 1.505 - 2.586  1.212 - 2.141 | <0.001  0.001 | 1.605  1.462 | 1.196 - 2.153  1.071 - 1.996 | 0.002  0.017 |
| ECOG-PS (PS 0-1 vs PS ≥2)  Histology (Non-Sq vs Sq) | 0.292  0.582 | 0.210 - 0.409  0.441 - 0.766 | <0.001  <0.001 | 0.412  0.649 | 0.285 - 0.596  0.483 - 0.872 | <0.001  0.004 |
| Stage (Recurrence vs IV)  PD-L1 (90-100% vs 50%-89%) | 0.596  0.770 | 0.450 - 0.789  0.579 - 1.024 | <0.001  0.072 | 0.560  0.682 | 0.369 - 0.850  0.508 - 0.915 | 0.006  0.011 |
| Brain metastasis (Yes vs No)  liver metastasis (Yes vs No)  CI therapy vs ICI monotherapy | 1.268  1.479  0.776 | 0.893 - 1.802  1.020 - 2.145  0.579 - 1.041 | 0.184  0.039  0.091 | 1.514  1.175  0.881 | 1.050 - 2.184  0.791 - 1.746  0.642 - 1.209 | 0.026  0.424  0.433 |

PFS, Progression free survival; OS, Overall survival; HR, Hazard ratio; AWGC, Asian Working Group for Cachexia; ECOG-PS, Eastern Cooperative Oncology Group performance status; Sq, Squamous cell carcinoma; CI, ChemoImmunotherapy; ICI, immune checkpoint inhibitor; PD-L1, Programmed Death Ligand-1
