# Supplementary material for: Identification of Hidden Cachexia Subgroup in PD‐L1‐High NSCLC: Comparative Analysis of the AWGC vs. Fearon Criteria
Source: J Cachexia Sarcopenia Muscle. 2026 Apr 12;17(2):e70281. doi: 10.1002/jcsm.70281 (PMC13070542; doi:10.1002/jcsm.70281)
Supplement: Supplementary file 5 — Table S3: Univariate and multivariate analyses of PFS and OS according to Fearon's cachexia criteria in patients without driver mutations. [file JCSM-17-e70281-s007.docx]

**Supplementary Table 3. Univariate and Multivariate Analyses of PFS and OS According to Fearon’s Cachexia Criteria in Patients Without Driver Mutations**

| Covariates | Crude HR | 95% CI | p-value | Adjusted HR | 95% CI | p-value |
| --- | --- | --- | --- | --- | --- | --- |
| Fearon’s cachexia vs No cachexia  Age (≥75 vs <75) | 1.555  1.099 | 1.128 - 2.144  0.854 - 1.415 | 0.007  0.464 | 1.544  1.059 | 1.110 - 2.146  0.808 - 1.389 | 0.009  0.677 |
| ECOG-PS (PS 0-1 vs PS ≥2)  Histology (Non-Sq vs Sq) | 0.411  0.828 | 0.301 - 0.561  0.645 - 1.063 | <0.001  0.139 | 0.471  0.846 | 0.338 - 0.657  0.652 - 1.098 | <0.001  0.209 |
| Stage (Recurrence vs IV)  PD-L1 (90-100% vs 50%-89%) | 0.792  0.635 | 0.587 - 1.070  0.495 - 0.813 | 0.129  <0.001 | 0.742  0.576 | 0.544 - 1.011  0.446 - 0.745 | 0.059  <0.001 |
| Brain metastasis (Yes vs No)  liver metastasis (Yes vs No)  CI therapy vs ICI monotherapy | 1.303  1.385  0.877 | 0.958 - 1.772  0.996 - 1.928  0.689 - 1.117 | 0.092  0.053  0.287 | 1.363  1.355  0.890 | 0.989 - 1.879  0.963 - 1.908  0.685 - 1.156 | 0.059  0.082  0.382 |

1. Univariate and Multivariate analysis for PFS

PFS, Progression free survival; OS, Overall survival; HR, Hazard ratio; AWGC, Asian Working Group for Cachexia; ECOG-PS, Eastern Cooperative Oncology Group performance status; Sq, Squamous cell carcinoma; CI, ChemoImmunotherapy; ICI, immune checkpoint inhibitor; PD-L1, Programmed Death Ligand-1

(B) Univariate and Multivariate analysis for OS

| Covariates | Crude HR | 95% CI | p-value | Adjusted HR | 95% CI | p-value |
| --- | --- | --- | --- | --- | --- | --- |
| Fearon’s cachexia vs No cachexia  Age (≥75 vs <75) | 2.041  1.611 | 1.435 - 2.903  1.212 - 2.141 | <0.001  0.001 | 1.999  1.395 | 1.391 - 2.874  1.023 - 1.903 | <0.001  0.035 |
| ECOG-PS (PS 0-1 vs PS ≥2)  Histology (Non-Sq vs Sq) | 0.292  0.582 | 0.210 - 0.409  0.441 - 0.766 | <0.001  <0.001 | 0.397  0.590 | 0.276 - 0.573  0.439 - 0.792 | <0.001  <0.001 |
| Stage (Recurrence vs IV)  PD-L1 (90-100% vs 50%-89%) | 0.596  0.770 | 0.450 - 0.789  0.579 - 1.024 | <0.001  0.072 | 0.487  0.681 | 0.321 - 0.739  0.507 - 0.915 | <0.001  0.011 |
| Brain metastasis (Yes vs No)  liver metastasis (Yes vs No)  CI therapy vs ICI monotherapy | 1.268  1.479  0.776 | 0.893 - 1.802  1.020 - 2.145  0.579 - 1.041 | 0.184  0.039  0.091 | 1.417  1.378  0.853 | 0.984 - 2.040  0.935 - 2.033  0.622 - 1.170 | 0.061  0.106  0.324 |

PFS, Progression free survival; OS, Overall survival; HR, Hazard ratio; AWGC, Asian Working Group for Cachexia; ECOG-PS, Eastern Cooperative Oncology Group performance status; Sq, Squamous cell carcinoma; PD-L1, Programmed Death Ligand-1; CI, ChemoImmunotherapy; ICI, immune checkpoint inhibitor
