# Supplementary material for: Identification of Hidden Cachexia Subgroup in PD‐L1‐High NSCLC: Comparative Analysis of the AWGC vs. Fearon Criteria
Source: J Cachexia Sarcopenia Muscle. 2026 Apr 12;17(2):e70281. doi: 10.1002/jcsm.70281 (PMC13070542; doi:10.1002/jcsm.70281)
Supplement: Supplementary file 6 — Table S4: Patients' characteristics (no‐cachexia vs. A‐only cachexia). [file JCSM-17-e70281-s006.docx]

**Supplementary Table 4**

**Patients' characteristics (No-cachexia vs A-only cachexia)**

| Characteristic | No-cachexia  n=230 | A-only cachexia  n=119 | *p-value* |
| --- | --- | --- | --- |
| Age, y  Median (range) | 71 [40-88] | 69 [36-90] | 0.625 |
| Sex  Male  Female | 177 (77.0)  53 (23.0) | 93 (78.2)  26 (21.8) | 0.893 |
| ECOG PS  0-1  **≥** 2 | 210 (91.3)  20 (8.7) | 100 (84.0)  19 (16.0) | 0.049 |
| BMI (kg/m^2^) | 23.01 [15.28-35.12] | 20.55 [13.97-26.91] | <0.001 |
| Body weight loss (%) | 0.0 [-9.0-27.0] | 1.0 [-3.0-22.0] | <0.001 |
| CRP (mg/dl) | 0.46 [0.0-26.0] | 2.81 [0.52-21.65] | <0.001 |
| Smoking history  Yes  No | 192 (83.5)  38 (16.5) | 105 (88.2)  14 (11.8) | 0.269 |
| Stage  IV  Postoperative recurrence | 174 (75.7)  56 (24.3) | 108 (90.8)  11 (9.2) | 0.001 |
| Histology  Squamous cell carcinoma  Adenocarcinoma  Others | 57 (24.8)  142 (61.7)  31 (13.5) | 45 (37.8)  56 (47.1)  18 (15.1) | 0.021 |
| Driver gene alteration  EGFR  ALK  ROS1 | 6 (2.6)  4 (1.7)  0 | 2 (1.7)  2 (1.7)  0 | 0.721  1.000  NA |
| Liver metastasis | 21 (9.1) | 28 (23.5) | 0.001 |
| Brain metastasis | 44 (19.1) | 12 (10.1) | 0.031 |
| Programmed cell death ligand 1, %  50-89  90-100 | 151 (65.7)  79 (34.3) | 72 (60.5)  47 (39.5) | 0.349 |
| Treatment regimen  Pembrolizumab monotherapy  Chemoimmunotherapy | 146 (63.5)  84 (36.5) | 72 (60.5)  47 (39.5) | 0.641 |

AWGC, Asian Working Group for Cachexia; A-only cachexia, AWGC-only cachexia; ECOG-PS, Eastern Cooperative Oncology Group performance status; BMI, Body mass index; EGFR, Epidermal Growth Factor Receptor; ALK, Anaplastic Lymphoma Kinase; ROS1, Receptor Oncogene Serine/threonine kinase 1
