# Supplementary material for: Identification of Hidden Cachexia Subgroup in PD‐L1‐High NSCLC: Comparative Analysis of the AWGC vs. Fearon Criteria
Source: J Cachexia Sarcopenia Muscle. 2026 Apr 12;17(2):e70281. doi: 10.1002/jcsm.70281 (PMC13070542; doi:10.1002/jcsm.70281)
Supplement: Supplementary file 7 — Table S5: Patients' characteristics (A‐only cachexia vs. A+F cachexia). [file JCSM-17-e70281-s001.docx]

**Supplementary Table 5**

**Patients' characteristics (A-only cachexia vs A+F cachexia)**

| Characteristic | A-only cachexia  n=119 | A+F cachexia  n=49 | p-value |
| --- | --- | --- | --- |
| Age, y  Median (range) | 69 [36-90] | 71 [48-85] | 0.568 |
| Sex  Male  Female | 93 (78.2)  26 (21.8) | 42 (85.7)  7 (14.3) | 0.294 |
| ECOG PS  0-1  **≥** 2 | 100 (84.0)  19 (16.0) | 32 (65.3)  17 (34.7) | 0.012 |
| BMI (kg/m^2^) | 20.55 [13.97-26.91] | 17.81 [14.44-19.93] | <0.001 |
| Body weight loss (%) | 1.0 [-3.0-22.0] | 8.0 [2.0-23.0] | <0.001 |
| CRP (mg/dl) | 2.8 [0.52-21.7] | 4.7 [0.5-19.4] | 0.017 |
| Smoking history  Yes  No | 105 (88.2)  14 (11.8) | 44 (89.8)  5 (10.2) | 1.000 |
| Stage  IV  Postoperative recurrence | 108 (90.8)  11 (9.2) | 43 (87.8)  6 (12.2) | 0.579 |
| Histology  Squamous cell carcinoma  Adenocarcinoma  Others | 45 (37.8)  56 (47.1)  18 (15.1) | 13 (26.5)  29 (59.2)  7 (14.3) | 0.315 |
| Driver gene alteration  EGFR  ALK  ROS1 | 2 (1.7)  2 (1.7)  0 | 4 (8.2)  0  0 | 0.061  1.000  NA |
| Liver metastasis | 28 (23.5) | 7 (14.3) | 0.214 |
| Brain metastasis | 12 (10.1) | 7 (14.3) | 0.432 |
| Programmed cell death ligand 1, %  50-89  90-100 | 72 (60.5)  47 (39.5) | 30 (61.2)  19 (38.8) | 1.000 |
| Treatment regimen  Pembrolizumab monotherapy  Chemoimmunotherapy | 72 (60.5)  47 (39.5) | 30 (61.2)  19 (38.8) | 1.000 |

AWGC, Asian Working Group for Cachexia; A-only cachexia, AWGC-only cachexia; A+F cachexia, AWGC + Fearon’s cachexia; ECOG-PS, Eastern Cooperative Oncology Group performance status; BMI, Body mass index; EGFR, Epidermal Growth Factor Receptor; ALK, Anaplastic Lymphoma Kinase; ROS1, Receptor Oncogene Serine/threonine kinase 1
