# Supplementary material for: Identification of Hidden Cachexia Subgroup in PD‐L1‐High NSCLC: Comparative Analysis of the AWGC vs. Fearon Criteria
Source: J Cachexia Sarcopenia Muscle. 2026 Apr 12;17(2):e70281. doi: 10.1002/jcsm.70281 (PMC13070542; doi:10.1002/jcsm.70281)
Supplement: Supplementary file 8 — Table S6: Comparison of diagnostic criteria components between A‐only cachexia vs. A+F cachexia. [file JCSM-17-e70281-s008.docx]

**Supplementary Table 6**

**Comparison of Diagnostic Criteria Components between A-only cachexia vs A+F cachexia**

| Characteristic | | A-only cachexia  n=119 | A+F cachexia  n=49 (%) | *p-value* |
| --- | --- | --- | --- | --- |
| BMI (kg/m^2^) | >=21 | 37 (31.1) | 0 | <0.001 |
|  | 20-20.9 | 42 (35.3) | 0 |  |
|  | <20 | 40 (33.6) | 49 (100%) |  |
| Body weight loss (%) | >=5% | 26 (21.8%) | 36 (73.5%) | <0.001 |
|  | 2%-4.9% | 23 (19.3%) | 13 (26.5%) |  |
|  | <2% | 70 (58.8%) | 0 |  |
| CRP (mg/dl) | >=0.5 | 119 (100%) | 49 (100%) | NA |
|  | <0.5 | 0 | 0 |  |
